# Scoping review of the association of plant-based diet quality with health outcomes

**DOI:** 10.3389/fnut.2023.1211535

**Published:** 2023-08-10

**Authors:** Richard M. Rosenfeld, Hailey M. Juszczak, Michele A. Wong

**Affiliations:** Department of Otolaryngology, SUNY Downstate Health Sciences University, Brooklyn, NY, United States

**Keywords:** vegan, vegetarian, plant-based diet, food frequency questionnaire, plant-based dietary index, diet quality, health outcomes, food as medicine

## Abstract

**Introduction:**

The association of plant-based dietary patterns with health outcomes has traditionally been assessed without considering nutritional value. The plant-based dietary index (PDI), first published in 2016, overcomes this limitation with both a healthful PDI (hPDI) and an unhealthful PDI (uPDI), based on the quality of plant foods consumed plus the frequency of animal foods. We sought to summarize the breadth of research using the hPDI and uPDI to gain insight into how the quality of plant-based dietary patterns might be associated with health outcomes.

**Methods:**

Scoping review of studies that used the PDI, hPDI, or uPDI to report associations with health outcomes. Multiple databases were searched from 2010 through April 2023 with 2 authors independently assessing eligibility and extracting data. In addition to assessing the association of the indices to health outcomes, we determined the frequency of concordant or discordant findings for hPDI versus PDI and for hPDI versus uPDI.

**Results:**

We included 95 articles (54% longitudinal, 37% cross-sectional, and 9% case–control) with a median sample size of 3,646. Higher hPDI levels were associated with favorable health outcomes in 36% of comparisons (most often for obesity, mortality, diabetes, cardiovascular disease, and psychiatric disorders), compared to 25% for the PDI and only 2% for the uPDI. Conversely, higher levels of the uPDI were associated with unfavorable health outcomes in 33% of comparisons, in contrast to under 1% for the hPDI and 2% for the PDI. When the hPDI association to an outcome was discordant with the uPDI or PDI, the significance and directionality always favored the hPDI over the uPDI, and nearly always favored the hPDI over the PDI.

**Discussion:**

Dietary indices that account for the quality of plant foods can show health benefits that might be missed by a generic plant-based index. A greater focus on the quality of plant foods could improve nutrition guidelines, raise awareness about the benefits of adding unrefined plant foods to the diet, and empower consumers to make incremental additions of such foods to displace unhealthy foods. We anticipate increasing use of indices that address food quality in future research.

## Introduction

Researchers often use food frequency questionnaires to assess how dietary patterns are associated with disease prevalence, incidence, and mortality. Resulting publications have traditionally used this information to create a simple dichotomy into diets with plant versus animal foods (e.g., vegetarian vs. omnivore), without considering the nutritional value of the plant foods consumed. This could potentially reduce, or obscure, any association of whole, fiber-rich healthy plant-foods with reduced disease incidence and mortality, compared to vegetarian diets with less healthy plant-foods, such as refined grains, processed foods, and sugar-sweetened snacks and beverages.

An example of the imprecision resulting from not addressing the overall quality of plant-based diets is the association of higher carbohydrate intake with increased mortality in a global health study (1). The investigators did not distinguish between whole-grain versus refined carbohydrates, making the results about carbohydrates overall difficult to interpret or generalize. Conversely, when carbohydrate quality is explicitly considered, a dose–response relationship is observed for whole-grain carbohydrates high in fiber and a reduced risk of mortality, type 2 diabetes, cardiovascular diseases, and colorectal and breast cancer (2). Similarly, consuming unrefined plant foods (e.g., nuts, fruits, vegetables and whole grains) can reduce the risk of stroke, heart failure, and coronary heart disease, whereas the opposite is seen for refined plant foods (e.g., refined grains and sugar-sweetened beverages) (3).

One solution to the impact of plant-based diet quality on health outcomes has been to calculate an overall *plant-based dietary index (PDI)* based on a food frequency questionnaire and to then stratify the PDI as a *healthful PDI* or an *unhealthful PDI* (Table 1) based on the type of plant foods consumed and the amount of animal foods (4). Plant foods considered healthy include nuts, fruits, legumes, vegetables and whole grains, whereas those considered less healthy include sweets, potatoes, refined grains, fruit juices, and sugar-sweetened beverages. Using this general approach to assessing diet quality, pooled analyses of cohort studies have shown the benefits of a healthful PDI for cardiovascular disease (5, 6), type 2 diabetes (4, 7), and weight reduction (8, 9). Others have used the term provegetarian dietary pattern (PVD), instead of PDI, for a similar classification into healthful versus unhealthful. Showing, for example, how the healthful PVD may reduce breast cancer risk (10).

**Table 1:** Composition of plant-based dietary indices.^*^

Plant-based index emphasis	Healthy plant foods	Less healthy plant foods	Animal foods
Overall PDI: higher intake of all plant foods and lower intake of all animal foods	Whole grains Fruits Vegetables Nuts Legumes Vegetable oils Tea/Coffee	Refined grains Fruit juices Potatoes Sweets/deserts Sugar-sweetened beverages	Meat Fish/seafood Animal fats Dairy Eggs Miscellaneous animal-based foods
Healthful PDI: higher intake of healthy plant foods and lower intakes of unhealthy plant foods and all animal foods			
Unhealthful PDI: higher intake of unhealthy plant foods and lower intakes of healthy plant foods and all animal foods			

PDI, plant-based dietary index. ^*^Food categories as defined by Satija et al. (4).

The advantage of considering plant-based diet quality when assessing health outcomes warrants a scoping review to map the available research evidence and to identify knowledge gaps (11). Although less common than systematic reviews, scoping reviews are increasing in popularity with established methodology and reporting standards (12–14). In contrast to systematic reviews, which synthesize quantitative evidence on the efficacy of an intervention for a specific condition, a scoping review offers primarily qualitative insight into a field of study through a broad, birds-eye view of a topic or subject area (13). Given the relatively recent distinction in the nutrition literature of healthy versus less healthy plant-based diets, we considered a scoping review ideal for exploring how this concept has influenced subsequent publications on the association of plant-based diets with health outcomes. Therefore, the goal of this scoping review is to highlight the importance of assessing plant-based diet quality so others can incorporate plant food quality into reviews, guidelines, and policies that associate diet with health outcomes.

## Methods

### Protocol

Our scoping review protocol was based on standards developed by JBI, the Joanna Briggs Institute, specifically for conducting a scoping review (13). The manuscript was structured in adherence to the Preferred Reporting Standards for Systematic Reviews and Meta-Analysis (PRISMA) extension for scoping reviews (14). The premise for this review is defined using the PICO criteria below for population, intervention, comparisons, and outcomes:

- Population: adults and children enrolled in studies comparing plant-based diet quality to health outcomes
- Intervention: dietary assessment using a food frequency questionnaire with categorization into an overall PDI or PVD, a healthful PDI or a healthful PVD (hPDI or hPVD), and an unhealthful PDI or an unhealthful PVD (uPDI or uPVD)
- Comparisons: when more than one index is reported, association with outcomes for the overall index versus the healthful index and for the healthful index versus the unhealthful index
- Outcomes: disease incidence, prevalence, or mortality as reported by the investigators, with hazard ratios for the highest dietary index level versus the lowest level (e.g., by quartiles, quintiles, deciles).

### Eligibility and search criteria

To be included in this review, the source article had to report original research assessing the association of a plant-based diet with a clinically relevant health outcome. The study design could be observational (e.g., cohort, case–control, or cross-sectional), experimental (e.g., clinical trial, randomized controlled trial), or population-based (e.g., national survey data) but must have included a healthful plant-based dietary index (hPDI or hPVD), an unhealthful index (uPDI or uPVD), or both. We excluded reviews, systematic reviews, meta-analyses, commentaries, case series, and correspondence (e.g., consensus reports).

Peer-reviewed articles meeting the above criteria, and addressing the PICO question above, were included if published between the period of January 2010 through April 2023, without language restrictions. Searches were performed with the assistance of an experienced information specialist in databases that included MEDLINE/PubMed, CINAHL, EMBASE, and Web of Science. The initial search strategy, drafted by an information specialist and refined through team discussion, was implemented in MEDLINE/Med, CINAHL, and EMBASE and used the terms “((healthy AND unhealthy) OR (healthful AND unhealthful)) AND (vegetarian OR vegan OR plant-based OR provegetarian OR pro-vegetarian OR plant-predominant).” Upon reviewing the initial search results we noted that some of the relevant articles cited publications that might also be relevant to our review, but instead of” unhealthy or unhealthful” used the terms “less healthy or less healthful.” We therefore updated the search with the expanded terms: ““((healthy AND unhealthy) OR (healthful AND unhealthful) OR (healthy AND “less healthy”) OR (healthful AND “less healthful”) OR (healthy AND overall) OR (healthful AND overall)) AND (vegetarian OR vegan OR plant-based OR provegetarian OR pro-vegetarian OR plant-predominant).”

### Selection of sources of evidence

To increase consistency, dual reviewers (HJM, MAW) screened articles for relevance, with disagreements on study selection and data extraction resolved by consensus and discussion, if needed. To reduce the possibility that articles were missed in the MEDLINE/Med, CINAHL, and EMBASE searches, a final search was performed in Web of Science for publications citing any of three key articles considered representative of source articles for the review (see Results for specific articles used).

A data-charting form for Excel was developed to extract all information from each source article, including information on authorship, article characteristics (publication year, country of origin, study aims or purpose), study sample (origin, size, demographics), sampling method (convenience, random, population cohort), sampling time frame (recruitment years), methodology (study design, food frequency questionnaire details), dietary classification (e.g., healthful unhealthful), follow-up information, outcomes assessed (including comparisons, such as by extreme quartiles, quintiles, or deciles), results (usually adjusted hazard ratios), and conclusions. We did not perform a risk of bias assessment for the included source articles because this is unnecessary in a scoping review (11) and is not part of the recommended reporting standards (14).

### Summary measures and results synthesis

We performed a descriptive and qualitative analysis, seeking to map the existing evidence and to highlight how considerations of plant-diet quality might impact associations with clinically important outcomes. We did not include quantitative data. Such as effect size, nor did we perform any data pooling using meta-analytic techniques, but we do include quantitative results for individual studies in the online Supplementary Appendix. Findings are reported using the PDI, which was used in 95% of studies, recognizing that this also includes a few studies that used the PVD. As consistent with scoping review methodology, we did formally test hypotheses using measures of statistical significance (12–14).

The primary outcome data from each source article was the association of the PDI, hPDI, and uPDI to each of the reported outcomes, as reflected by comparing the highest level (quartile, quintile, or decile) of each index to the lowest level. An association was termed “favorable” if both statistically significant and a higher index level correlated with a better health outcome (e.g., less disease, lower mortality, better cardiometabolic marker). Conversely, an association was termed “unfavorable” if both statistically significant and a higher index level correlated with a worse health outcome (e.g., more disease, higher mortality, adverse cardiometabolic marker). We did not judge any statistically non-significant associations as favorable versus unfavorable, nor did we seek to make direct statistical comparisons between different indices.

We further assessed secondary outcomes on the concordance, or discordance, of the associations for the 3 indices (PDI, hPDI, uPDI) by comparing the statistical significance and directionality of the relationship to outcome in a specific study. This was used to classify comparisons between hPDI versus PDI and between hPDI versus uPDI as “favors hPDI,” “both same,” or “favors” comparator (PDI or uPDI):

- Favors hPDI: when comparing the hPDI to the PDI (or uPDI) the comparison “favors hPDI” if the hPDI had a significantly favorable HR and the PDI (or uPDI) had a non-significant HR, or if the hPDI had a non-significant HR but the PDI (or uPDI) had significantly unfavorable HR. In both cases the HRs were discordant and the hPDI did “better” than the comparator, leading a result that “favors hPDI.”
- Both same: when comparing the hPDI to the PDI the comparisons was deemed “both same” if the HRs for each index were concordant: both significantly favorable, both non-significant, or both significantly non-favorable. The same criteria applied to comparing the hPDI to the uPDI.
- Favors PDI: when comparing the hPDI to the PDI the comparison “favors PDI” if the PDI had a significantly favorable HR and the hPDI was non-significant, or if the PDI had a non-significant HR but the hPDI had significantly unfavorable HR. In both cases the HRs were discordant and the PDI did “better” than the PDI, leading a result that “favors PDI.”
- Favors uPDI: when comparing the hPDI to the uPDI the comparison “favors uPDI” if the uPDI had a significantly favorable HR and the hPDI was non-significant, or if the uPDI had a non-significant HR but the hPDI had significantly unfavorable HR. In both cases the HRs were discordant and the uPDI did “better” than the uPDI, leading a result that “favors uPDI.”

The above comparisons are reported for studies contributing to specific outcome (e.g., all-cause mortality, hypertension, metabolic syndrome) and were also combined for all comparisons and outcomes to give a global perspective of how stratifying a plant-based diet based on food quality might impact associations.

## Results

**Figure 1:**
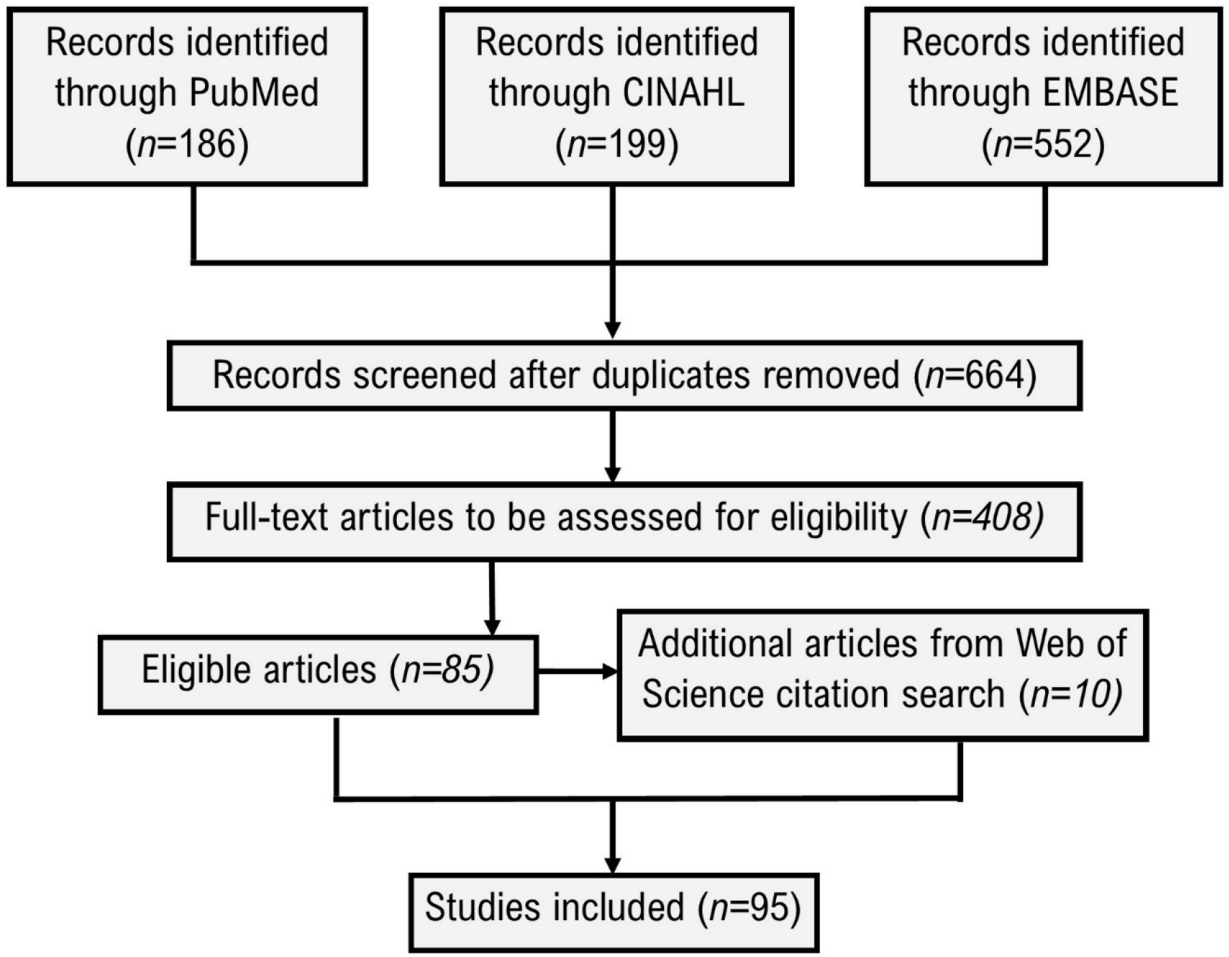
Selection of evidence sources for scoping review.

The literature search (Figure 1) identified 95 source articles (10, 15–108), for which full details of data extraction can be found in the online Supplementary Appendix. The Web of Science search was based on citations of three articles (23, 46, 87), published between 2017 and 2019, identified in the prior searches and considered representative of those sought for the review. Articles were published between 2017 and 2023 with a median sample size of 3,646, ranging from 22 to 592,571, and with upper and lower quartiles of 456 and 14,568, respectively. The countries of origin for the source articles were United States (*n* = 30 publications), Iran (*n* = 20), Korea (*n* = 9), China (*n* = 8), Spain (*n* = 8), United States/United Kingdom (*n* = 3), Australia (*n* = 3), Germany (*n* = 3), Singapore (*n* = 3), Saudi Arabia (*n* = 2), Greece (*n* = 2), France (*n* = 2), Japan (*n* = 1), and Belgium (*n* = 1).

All studies were observational, with a longitudinal (cohort) design for 54%, cross-sectional for 37%, case–control format for 9%. The PDI was used in 95% of studies, with only 5 studies reporting PVG as the primary outcome (10, 21, 38, 78, 79). The index combinations were PDI/hPDI/uPDI in 69 studies, PDI/hPDI in 9, hPDI/uPDI in 7, PVG/hPVG/uPVG in 5, PDI/hPDI/uPDI/PVG in 3, and hPDI only in 2. The primary outcomes were obesity in 21 studies, cardiometabolic risk factors in 20, mortality in 10, diabetes in 9, psychiatric disorders in 9, men’s health in 8, cardiovascular disease in 7, breast cancer in 5, inflammation in 5, chronic kidney disease in 3, sleep quality in 3, quality of life in 2, bone biomarkers in 2, and 1 each for asthma, glioma, fecundability, COVID-19, micronutrients, and infant growth.

The hPDI demonstrated the most frequent association with favorable health outcomes (Table 2), with 36.2% having significantly more favorable results (*p* < 0.05, as reported by the investigators) when comparing the highest hPDI level to the lowest. Conversely, higher uPDI levels were associated with unfavorable health outcomes for 32.5% of comparisons. The hPDI was almost never (0.4%) associated with unfavorable outcomes and the uPDI was rarely associated (2.0%) with favorable outcomes. Although 24.6% of higher PDI levels were associated with favorable health outcomes, the hPDI was about 50% more likely to demonstrate this type of relationship (36.2% vs. 24.6%).

**Table 2:** Association of plant-based indices with health outcomes, showing the frequency of association type (favorable, unfavorable, or nonsignificant) for each index (hPDI, PDI, or uPDI).

Association^*^	hPDI, *n* = 268^†^ (%)	PDI, *n* = 260^†^ (%)	uPDI, *n* = 249^†^ (%)
Significant favorable association	97 (36.2)	64 (24.6)	5 (2.0)
Significant unfavorable association	1 (0.4)	1 (0.4)	81 (32.5)
Nonsignificant association	170 (63.4)	195 (75.0)	163 (65.5)

PDI, overall plant-based dietary index; hPDI, healthful PDI; uPDI, unhealthful PDI. ^*^Association with outcome was significant if *p* < 0.05, favorable if the highest level of the index (e.g., quartile, quintile, decile) was associated with a more favorable outcome than the lowest level, unfavorable if associated with a more unfavorable outcome. ^†^Number of comparisons for the specific index, which exceeds the number of studies (95) because of multiple comparisons in most studies.

When discordant associations (Table 3) were observed for the hPDI versus a comparator (PDI or uPDI), the results most often favored the hPDI (23.0%) over the PDI and always favored the hPDI (52.3%) over the uPDI. Concordant associations were most often observed for the hPDI versus PDI (70.4%) with a minority of hPDI versus uPDI associations showing concordance (47.7%). For all comparisons combined, 59.6% had concordant results, 36.9% were discordant favoring the hPDI, and only 3.5% were discordant favoring the comparator (PDI or uPDI).

**Table 3:** Concordant versus discordant comparisons, showing the frequency of comparison outcome (discordant favoring hPDI, concordant, or discordant favoring comparator) for hPDI versus comparators (PDI or uPDI).

Comparison	Discordant, favoring hPDI (%)	Concordant^*^ (%)	Discordant, favoring comparator (%)
hPDI versus PDI, *N* = 213	49 (23.0)	150 (70.4)	14 (6.6)
hPDI versus uPDI, *N* = 193	101 (52.3)	92 (47.7)	0 (0)
Combined, *N* = 406	150 (36.9)	24 (59.6)	14 (3.5)

PDI, plant-based dietary index; hPDI, healthful PDI; uPDI, unhealthful PDI. ^*^Concordant: hPDI and comparator have same statistical significance (e.g., significant vs. non-significant) and directionality (positive vs. negative association with outcome).

For the outcomes in Table 4 with at least 10 comparisons of the highest versus lowest index levels, the most frequent significantly favorable associations with the hPDI were found for psychiatric disorders (94% of comparisons), diabetes (64%), cardiovascular disease (45%), mortality (43%), and obesity (42%). The most frequent significantly unfavorable associations with the uPDI were found for psychiatric disorders (50%), mortality (39%), obesity (36%), and cardiometabolic risk factors (31%). The hPDI level was more frequently associated with favorable outcomes than the uPDI was associated with unfavorable outcomes for psychiatric disorders (94% vs. 50%), diabetes (64% vs. 9%), and cardiovascular disease (45% vs. 9%).

**Table 4:** Favorable versus unfavorable plant-based index associations with specific outcomes.

N^†^	Outcome		Statistically significant associations of plant-based indices to outcome^*^						Studies (references)
			Favorable			Unfavorable			
		Tests^#^	hPDI (%)	PDI	uPDI	hPDI	PDI	uPDI (%)	
21	Obesity	36	15(42)	9	0	0	0	13 (36)	(24, 29, 34, 38, 42, 47, 48, 55, 60, 65–67, 69, 72, 78, 83, 88, 91, 98, 99, 105)
20	Cardiometabolic risk factors	77	12(16)	11	3	1	0	24 (31)	(16, 19, 26, 34, 40, 47–49, 51, 57, 58, 65, 66, 76, 88, 90, 91, 99, 100)
10	Mortality^.^	23	10(43)	10	0	0	0	9 (39)	(20, 23, 35, 44, 46, 50, 59, 82, 95, 101)
9	Diabetes	11	7(64)	4	0	0	0	1 (9)	(24, 29, 30, 36, 53, 57, 89, 96, 106)
9	Psychiatric disorders	16	15 (94)	8	0	0	0	8 (50)	(18, 32, 39, 61, 63, 73, 103, 105, 107)
8	Men’s health	23	3(13)	1	0	0	1	2 (9)	(28, 56, 64, 66, 67, 75, 77, 104)
7	Cardiovascular disease	11	5(45)	2	0	0	0	1 (9)	(25, 41, 44, 54, 76, 87, 101)
5	Breast cancer	13	3 (23)	5	0	0	0	2 (15)	(10, 79, 84–86)
5	Inflammation	9	3 (33)	1	0	0	0	0 (0)	(17, 24, 27, 81, 89)
4	Gastrointestinal cancer	7	6(86)	5	1	0	0	3 (43)	(52, 79, 97, 102)
4	Quality of life	5	5(100)	3	0	0	0	5 (100)	(23, 68, 93, 108)
3	Chronic kidney disease	9	1 (11)	2	0	0	0	3 (33)	(45, 70, 94)
3	Sleep quality	3	2 (66)	0	0	0	0	3 (100)	(32, 43, 81)
2	Bone biomarkers	6	2(33)	1	0	0	0	2 (33)	(37, 89)
1	Glioma	1	1 (100)	1	0	0	0	1 (100)	(74)
1	Fecundability	1	1 (100)	0	0	0	0	1 (100)	(62)
1	COVID-19	3	3 (100)	N/A	N/A	0	N/A	N/A	(71)
1	Infant growth	9	0 (0)	0	0	0	0	1 (11)	(33)
1	Micronutrients	2	1(50)	2	0	0	0	2 (100)	(21)
1	Asthma	3	1(33)	1	1	0	0	0 (0)	(15)

PDI, plant-based dietary index; hPDI healthful PDI; uPDI unhealthful. ^†^Number of source articles reporting the outcome. ^#^Total number of statistical tests comparing highest versus lowest index level; percentages shown for favorable hPDI and unfavorable uPDI associations are based on this number as the denominator. ^*^Comparison of highest level (quartile, quintile, or decile) to lowest level.

Tables 5–13 show the frequency of concordant versus discordant plant-based index comparisons by specific outcome studied. In Table 5, mortality, comparisons of the hPDI versus PDI are largely concordant, but some of the outcomes for hPDI versus uPDI are often discordant, as seen for all-cause mortality and cardiovascular disease mortality. A similar pattern is seen in Table 7 for psychiatric disorders (anxiety, cognitive impairment, and depression) and in Table 11 for obesity (fatty liver disease, visceral adiposity, central obesity, general obesity, and overweight or obese). Specific outcomes in other tables also show discordance that favors the hPDI over the uPDI, including Table 6 (hypertension, metabolic syndrome, and HDL cholesterol), Table 8 (breast cancer and colorectal cancer), Table 9 (serum insulin and type 2 diabetes), and Table 13 (sleep quality index).

**Table 5:** Mortality and cardiovascular diseases, concordant versus discordant plant-based index comparisons.

Outcome	*N*	hPDI versus PDI statistical significance with outcome			hPDI versus uPDI statistical significance with outcome			Studies (references)
		Favors hPDI^*^	Both same^†^	Favors PDI^‡^	Favors hPDI^*^	Both same^†^	Favors uPDI^‡^	
All-cause mortality	10	1	6	2	5	5	0	(20, 23, 35, 44, 46, 50, 59, 82, 95, 101)
Cardiovascular disease mortality	7	0	6	0	5	2	0	(25, 35, 41, 44, 54, 87, 101)
Cancer mortality	4	0	2	2	1	3	0	(23, 50, 59, 95)
Breast cancer mortality	1	0	1	0	0	1	0	(20)
Non-breast cancer mortality	1	1	0	0	1	0	0	(20)
Coronary heart disease	2	1	1	0	1	1	0	(87, 101)
Cardiovascular disease	4	1	2	1	2	2	0	(44, 54, 76, 101)
Stroke	2	1	1	0	1	1	0	(25, 101)

PDI, plant-based dietary index; hPDI, healthful PDI; uPDI, unhealthful PDI. ^*^Favors hPDI if hPDI hazard ratio statistical significance for the outcome is more favorable than the comparator (PDI or uPDI): e.g., hPDI significantly favorable and comparator non-significant or unfavorable, or hPDI non-significant and comparator unfavorable. ^†^Both same if hPDI hazard ratio has same relationship to outcome as comparator (both significantly favorable, both non-significant, or both significantly unfavorable). ^‡^Favors comparator (PDI or uPDI) if hazard ratio statistical significance for the outcome is more favorable than the hPDI: e.g., comparator significantly favorable and hPDI non-significant or unfavorable, or comparator non-significant and hPDI unfavorable.

**Table 6:** Cardiometabolic risk factors, concordant versus discordant plant-based index comparisons.

Outcome	*N*	hPDI versus PDI statistical significance with outcome			hPDI versus uPDI statistical significance with outcome			Studies (references)
		Favors hPDI^*^	Both same^†^	Favors PDI^‡^	Favors hPDI^*^	Both same^†^	Favors uPDI^‡^	
Hypertension	5	1	4	0	4	1	0	(48, 49, 51, 57, 88)
HDL cholesterol	8	0	8	0	8	6	0	(26, 34, 48, 65, 78, 90, 91, 99)
LDL cholesterol	5	0	5	0	0	4	1	(26, 34, 65, 90, 99)
Lip accumulation product	1	0	1	0	0	1	0	(88)
Metabolic syndrome	5	1	4	0	4	1	0	(19, 47, 48, 66, 76)
Non-HDL	1	0	1	0	0	1	0	(90)
Systolic blood pressure	4	1	3	0	1	3	0	(15, 34, 65, 78)
Diastolic blood pressure	4	2	2	0	1	3	0	(16, 34, 65, 78)
Trimethylamine oxide	1	0	0	1	0	0	1	(40)
Total cholesterol	5	1	4	0	1	4	0	(34, 65, 90, 91, 99)
Total cholesterol/HDL	1	1	1	0	1	1	0	(65, 99)
Triglycerides, high	7	0	7	0	2	5	0	(26, 34, 48, 65, 88, 90, 91)
Triglyceride-glucose index	1	0	1	0	0	1	0	(88)
Triglyceride/HDL	1							(65)
Weight	1	0	0	1	0	1	0	(26)
Waist circumference	4	0	4	0	1	3	0	(34, 78, 91, 99)
Waist, hyper-triglycemic	1	0	1	0	0	1	0	(91)
Glucose, high	5	0	3	1	1	4	0	(26, 34, 48, 65, 78, 88)

HDL, high-density lipoprotein; LDL, low-density lipoprotein; PDI, plant-based dietary index; hPDI, healthful PDI; uPDI, unhealthful PDI. ^*^Favors hPDI if hPDI hazard ratio statistical significance for the outcome is more favorable than the comparator (PDI or uPDI): e.g., hPDI significantly favorable and comparator non-significant or unfavorable, or hPDI non-significant and comparator unfavorable. †Both same if hPDI hazard ratio has same relationship to outcome as comparator (both significantly favorable, both non-significant, or both significantly unfavorable). ^‡^Favors comparator (PDI or uPDI) if hazard ratio statistical significance for the outcome is more favorable than the hPDI: e.g., comparator significantly favorable and hPDI non-significant or unfavorable, or comparator non-significant and hPDI unfavorable.

**Table 7:** Psychiatric disorders, concordant versus discordant plant-based index comparisons.

Outcome	*N*	hPDI versus PDI statistical significance with outcome			hPDI versus uPDI statistical significance with outcome			Studies (references)
		Favors hPDI^*^	Both same^†^	Favors PDI^‡^	Favors hPDI^*^	Both same^†^	Favors uPDI^‡^	
Anxiety	3	1	2	0	3	0	0	(32, 73, 105)
Cognitive impairment	4	2	3	0	0	1	3	(61, 63, 103, 107)
Depression	3	1	2	0	3	0	0	(32, 73, 105)
Stress	4	3	1	0	4	0	0	(18, 32, 73, 105)
Impulsivity	1	1	0	0	1	0	0	(39)

PDI, plant-based dietary index; hPDI, healthful PDI; uPDI, unhealthful PDI. ^*^Favors hPDI if hPDI hazard ratio statistical significance for the outcome is more favorable than the comparator (PDI or uPDI): e.g., hPDI significantly favorable and comparator non-significant or unfavorable, or hPDI non-significant and comparator unfavorable. ^†^Both same if hPDI hazard ratio has same relationship to outcome as comparator (both significantly favorable, both non-significant, or both significantly unfavorable). ^‡^Favors comparator (PDI or uPDI) if hazard ratio statistical significance for the outcome is more favorable than the hPDI: e.g., comparator significantly favorable and hPDI non-significant or unfavorable, or comparator non-significant and hPDI unfavorable.

**Table 8:** Cancer incidence, concordant versus discordant plant-based index comparisons.

Outcome	*N*	hPDI versus PDI statistical significance with outcome			hPDI versus uPDI statistical significance with outcome			Studies (references)
		Favors hPDI^*^	Both same^†^	Favors PDI^‡^	Favors hPDI^*^	Both same^†^	Favors uPDI^‡^	
Prostate cancer, total	1	0	1	0	NS	NS	NS	(64)
Breast cancer	5	1	4	0	2	3	0	(80, 84–86)
Breast cancer, recurrence	1	0	1	0	0	1	0	(20)
Colorectal cancer	3	0	3	0	3	1	0	(52, 97, 102)
Esophageal cancer	1	0	1	0	1	0	0	(79)
Stomach cancer	1	0	1	0	1	0	0	(79)
Pancreatic cancer	1	0	1	0	1	0	0	(79)

PDI, plant-based dietary index; hPDI, healthful PDI; uPDI, unhealthful PDI. ^*^Favors hPDI if hPDI hazard ratio statistical significance for the outcome is more favorable than the comparator (PDI or uPDI): e.g., hPDI significantly favorable and comparator non-significant or unfavorable, or hPDI non-significant and comparator unfavorable. ^†^Both same if hPDI hazard ratio has same relationship to outcome as comparator (both significantly favorable, both non-significant, or both significantly unfavorable). ^‡^Favors comparator (PDI or uPDI) if hazard ratio statistical significance for the outcome is more favorable than the hPDI: e.g., comparator significantly favorable and hPDI non-significant or unfavorable, or comparator non-significant and hPDI unfavorable.

**Table 9:** Diabetes, concordant versus discordant plant-based index comparisons.

Outcome	*N*	hPDI versus PDI statistical significance with outcome			hPDI versus uPDI statistical significance with outcome			Studies (references)
		Favors hPDI^*^	Both same^†^	Favors PDI^‡^	Favors hPDI^*^	Both same^†^	Favors uPDI^‡^	
Gestational diabetes	2	0	1	1	0	2	0	(31, 106)
HOMA-IR	1	1	0	0	1	0	0	(89)
QUICKI	1	0	1	0	0	1	0	(89)
Serum insulin	2	1	0	1	2	0	0	(24, 89)
Type 2 diabetes	5	2	3	0	4	0	0	(30, 36, 53, 57, 96)
Hemoglobin A1c	1	0	1	0	0	1	0	(26)

HOMA-IR, homeostatic model assessment of insulin resistance; PDI, plant-based dietary index; hPDI, healthful PDI; uPDI, unhealthful PDI; QUICKI, quantitative insulin sensitivity check index. ^*^Favors hPDI if hPDI hazard ratio statistical significance for the outcome is more favorable than the comparator (PDI or uPDI): e.g., hPDI significantly favorable and comparator non-significant or unfavorable, or hPDI non-significant and comparator unfavorable. ^†^Both same if hPDI hazard ratio has same relationship to outcome as comparator (both significantly favorable, both non-significant, or both significantly unfavorable). ^‡^Favors comparator (PDI or uPDI) if hazard ratio statistical significance for the outcome is more favorable than the hPDI: e.g., comparator significantly favorable and hPDI non-significant or unfavorable, or comparator non-significant and hPDI unfavorable.

**Table 10:** Inflammation and inflammatory markers, concordant versus discordant plant-based index comparisons.

Outcome	*N*	hPDI versus PDI statistical significance with outcome			hPDI versus uPDI statistical significance with outcome			Studies (references)
		Favors hPDI^*^	Both same^†^	Favors PDI^‡^	Favors hPDI^*^	Both same^†^	Favors uPDI^‡^	
High-sensitivity C-reactive protein	5	1	3	1	1	4	0	(17, 24, 27, 81, 89)
Interleukin 1 beta	1	0	1	0	0	1	0	(81)
Interleukin 6	1	0	1	0	0	1	0	(24)
Transforming growth factor beta	2	0	1	1	0	1	0	(27, 81)
osteocalcin	1	0	1	0	1	0	0	(89)
Human C-telopeptide of type 1 collagen	1	1	0	0	1	0	0	(89)
25-hydroxy vitamin D	1	0	1	0	0	1	0	(89)
Parathyroid hormone	1	0	0	1	0	1	0	(89)

PDI, plant-based dietary index; hPDI, healthful PDI; uPDI, unhealthful PDI. ^*^Favors hPDI if hPDI hazard ratio statistical significance for the outcome is more favorable than the comparator (PDI or uPDI): e.g., hPDI significantly favorable and comparator non-significant or unfavorable, or hPDI non-significant and comparator unfavorable. ^†^Both same if hPDI hazard ratio has same relationship to outcome as comparator (both significantly favorable, both non-significant, or both significantly unfavorable). ^‡^Favors comparator (PDI or uPDI) if hazard ratio statistical significance for the outcome is more favorable than the hPDI: e.g., comparator significantly favorable and hPDI non-significant or unfavorable, or comparator non-significant and hPDI unfavorable.

**Table 11:** Obesity, concordant versus discordant plant-based index comparisons.

Outcome	*N*	hPDI versus PDI statistical significance with outcome			hPDI versus uPDI statistical significance with outcome			Studies (references)
		Favors hPDI^*^	Both same^†^	Favors PDI^‡^	Favors hPDI^*^	Both same^†^	Favors uPDI^‡^	
Adiponectin	1	1	0	0	1	0	0	(24)
Adiposity, subcutaneous	1	0	1	0	0	1	0	(83)
Adiposity, visceral	2	1	1	0	2	0	0	(83, 88)
Alanine transaminase	1	0	1	0	1	0	0	(69)
Alkaline phosphatase	1	0	1	0	0	1	0	(69)
Aspartate transaminase	1	0	1	0	1	0	0	(69)
Body mass index	6	0	5	1	0	4	0	(65–67, 78, 91, 99)
Body mass index ≥30	1	0	1	0	0	1	0	(34)
Leptin	1	1	0	0	1	0	0	(24)
Leptin, free index	1	1	0	0	1	0	0	(24)
Leptin, soluble receptor	1	1	0	0	1	0	0	(24)
Liver disease, fatty	4	1	2	1	1	3	0	(55, 60, 69, 83)
Liver signal intensity	1	0	1	0	0	1	0	(83)
Liver, fatty index	1	0	1	0	1	0	0	(68)
MUO by IDF criteria	1	1	0	0	1	0	0	(72)
MUO by HOMA-IR criteria	1	1	0	0	1	0	0	(72)
Obesity, central	5	0	5	0	2	2	1	(29, 42, 47, 88, 105)
Obesity, general	2	1	1	0	2	0	0	(88, 98)
Overweight or obese	2	0	2	0	2	0	0	(38, 105)
Retinol binding protein 4	1	0	1	0	0	1	0	(24)

HOMA-IR, Homeostatic model assessment of insulin resistance; IDF, International Diabetes Federation; MUO, metabolically unhealthy obestity phenotype; PDI, plant-based dietary index; hPDI, healthful PDI; uPDI, unhealthful PDI. ^*^Favors hPDI if hPDI hazard ratio statistical significance for the outcome is more favorable than the comparator (PDI or uPDI): e.g., hPDI significantly favorable and comparator non-significant or unfavorable, or hPDI non-significant and comparator unfavorable. ^†^Both same if hPDI hazard ratio has same relationship to outcome as comparator (both significantly favorable, both non-significant, or both significantly unfavorable). ^‡^Favors comparator (PDI or uPDI) if hazard ratio statistical significance for the outcome is more favorable than the hPDI: e.g., comparator significantly favorable and hPDI non-significant or unfavorable, or comparator non-significant and hPDI unfavorable.

**Table 12:** Infant growth, concordant versus discordant plant-based index comparisons.

Outcome	*N*	hPDI versus PDI statistical significance with outcome			hPDI versus uPDI statistical significance with outcome			Studies (references)
		Favors hPDI^*^	Both same^†^	Favors PDI^‡^	Favors hPDI^*^	Both same^†^	Favors uPDI^‡^	
Overweight at 2 m	1	0	1	0	0	1	0	(33)
Overweight at 4 m	1	0	1	0	0	1	0	(33)
Underweight at 2 m	1	0	1	0	0	1	0	(33)
Stunted at 2 m	1	0	1	0	0	1	0	(33)
Stunted at 4 m	1	0	1	0	1	0	0	(33)
Microcephaly at 2 m	1	0	1	0	0	1	0	(33)
Microcephaly at 4 m	1	0	1	0	0	1	0	(33)
Macrocephaly at 2 m	1	0	1	0	0	1	0	(33)
Macrocephaly at 4 m	1	0	1	0	0	1	0	(33)

PDI, plant-based dietary index; hPDI, healthful PDI; uPDI, unhealthful PDI. ^*^Favors hPDI if hPDI hazard ratio statistical significance for the outcome is more favorable than the comparator (PDI or uPDI): e.g., hPDI significantly favorable and comparator non-significant or unfavorable, or hPDI non-significant and comparator unfavorable. ^†^Both same if hPDI hazard ratio has same relationship to outcome as comparator (both significantly favorable, both non-significant, or both significantly unfavorable). ^‡^Favors comparator (PDI or uPDI) if hazard ratio statistical significance for the outcome is more favorable than the hPDI: e.g., comparator significantly favorable and hPDI non-significant or unfavorable, or comparator non-significant and hPDI unfavorable.

**Table 13:** Miscellaneous outcomes, concordant versus discordant plant-based index comparisons.

Outcome	*N*	hPDI versus PDI statistical significance with outcome			hPDI versus uPDI statistical significance with outcome			Studies (references)
		Favors hPDI^*^	Both same^†^	Favors PDI^‡^	Favors hPDI^*^	Both same^†^	Favors uPDI^‡^	
Chronic kidney disease	1	1	0	0	1	0	0	(45)
Glioma	1	0	1	0	1	0	0	(74)
Endothelial dysfunction	2	0	4	0	NS	NS	NS	(65, 67)
Erectile dysfunction	3	1	2	0	NS	NS	NS	(28, 65, 67)
Fecundability	1	1	0	0	1	0	0	(62)
PSA, elevated	1	1	0	0	NS	NS	NS	(75)
Severe COVID	1	1	NS	NS	0	NS	NS	(71)
Sleep, later chronotype	1	NS	NS	NS	1	0	0	(43)
Sleep, quality index	2	1	1	0	2	0	0	(32, 81)
Testosterone, total	3	0	3	0	NS	NS	NS	(55, 65, 67)
Quality of life, healthy aging	1	0	1	0	NS	NS	NS	(108)
Quality of life, physical score	1	0	1	0	1	0	0	(22)
Quality of life, mental score	1	0	1	0	1	0	0	(22)

PDI, plant-based dietary index; hPDI, healthful PDI; uPDI, unhealthful PDI. ^*^Favors hPDI if hPDI hazard ratio statistical significance for the outcome is more favorable than the comparator (PDI or uPDI): e.g., hPDI significantly favorable and comparator non-significant or unfavorable, or hPDI non-significant and comparator unfavorable. ^†^Both same if hPDI hazard ratio has same relationship to outcome as comparator (both significantly favorable, both non-significant, or both significantly unfavorable). ^‡^Favors comparator (PDI or uPDI) if hazard ratio statistical significance for the outcome is more favorable than the hPDI: e.g., comparator significantly favorable and hPDI non-significant or unfavorable, or comparator non-significant and hPDI unfavorable.

## Discussion

The aim of our scoping review was to highlight the importance of assessing plant-based diet quality, beyond using “plant-based” as an umbrella term (e.g., vegan, vegetarian), when assessing the association of diet type with health outcomes. We found a robust, and rapidly growing, body of literature that investigates how the quality and nutritional value of a plant-based diet is positively associated with health outcomes. The 95 studies we identified, most published in 2021 or later, represent diverse population cohorts from investigators in the United States, Western Europe, Middle East, Asia, and Australia. The diverse outcomes (Table 4) are most often related to the broad topics of obesity, cardiometabolic risk factors, overall- and disease-specific mortality, diabetes, cardiovascular disease, psychiatric disorders, men’s health, and cardiovascular disease.

For 33 to 36% of comparisons (Table 2), the highest levels of the hPDI and uPDI are associated with favorable and unfavorable health outcomes, respectively, whereas the highest PDI levels have favorable associations in only 25% of comparisons. Moreover, when the index associations are discordant (Table 3), the hPDI is more favorably associated with outcomes than the uPDI in 52% of comparisons and the hPDI is more favorably associated with outcomes than the PDI in 23% of comparisons. In aggregate, these findings show that stratifying the PDI into healthful versus unhealthful indices is superior to the PDI alone in assessing how plant-based diets are associated with health outcomes.

Our findings also identify some gaps in the existing knowledge base. For example, we did not identify any studies from investigators in Africa, South America, Central America, Scandinavia, or Eastern Europe, which raises concerns about generalizability, potentially to resource-challenged countries and regions. There is also limited information on how plant-based diet quality is associated with many clinical outcomes, based on conditions not listed in Table 4 and on those with only a few source articles (e.g., COVID-19, quality of life, sleep quality, fecundability, infant growth, glioma, bone biomarkers, and some cancers). Even when there are many source articles in an outcome category, more comparable outcomes may only be covered in 1 or 2 studies (Tables 5–13) and the measures used are heterogeneous.

The gaps and heterogeneity noted help to explain why relatively few meta-analyses have been performed using not just PDI, but also hPDI and uPDI. In all published reviews, however, where this distinction has been made, 4–6,9 the investigators find significant quantitative benefits related to diet quality, consistent with our qualitative and descriptive findings. This work builds upon a precursor concept of assessing mortality with a provegetarian food pattern, emphasizing plant-derived foods of any quality, in contrast to broad dietary classifications as vegan, vegetarian, or omnivore. Satija et al. (4) in 2016 ushered in the current focus on healthful versus unhealthful plant-based indices, when they showed substantially lower risk of developing type 2 diabetes with a diet rich in high-quality plant foods (Table 1), and a lower intake of animal foods and less healthy plant foods.

A benefit of defining a dietary pattern based on the frequency of healthy plant-foods consumed is the ability to study large populations using continuous indices (PDI, hPDI, uPDI), based on dietary assessment data to evaluate the relative quality of individuals’ dietary intakes (9). Moreover, these indices often identify benefits of healthy plant foods that might be missed when using a single overall measure of plant foods in the diet (Tables 2–4). Plant-based diet indices overcome limitations of discrete dietary categories because they align with the continuum of plant-forward, flexitarian, diets that exist in real-world settings. Further, the goal of increasing healthy plant foods in a diet, as opposed to restricting animal foods, is not only appealing but aligns with research showing that mortality may be driven more by the paucity of healthy plant foods (e.g., whole grains, fruits, nuts/seeds, legumes) than by the excess of meat (red and processed) and unhealthy plant foods (e.g., sugar-sweetened beverages) (109).

Differentiating between healthy versus less healthy aspects of plant-based diets has significant implications for researchers, policy makers (e.g., clinical practice guideline developers), and for consumers and the public (Table 14). Beyond focusing on overall diet quality, the healthfulness of individual foods has also received increased attention with nutrient profiling systems such as the Food Compass, which assigns a score from 1 (least healthful) to 100 (most healthful) based on 54 attributes across 9 domains: nutrient ratios, vitamins, minerals, food ingredients, additives, processing, specific lipids, fiber and protein, and phytochemicals (110). The healthy plant foods in Table 1 receive high scores on the Food Compass (110), which is associated with optimal cardiometabolic health and lower all-cause mortality (111). In contrast, the less healthy plant-based foods and the animal foods in Table 1 receive much lower scores.

**Table 14:** Benefits of using the healthful and unhealthful plant-based dietary index in nutrition research.

Benefit of classifying PDI in terms of hPDI and uPDI	Implications for researchers	Implication for policy makers	Implication for public/consumers
Can calculate healthful and unhealthful indices from preexisting dietary intake data	Facilitates research because no new data required to calculate PDI, hPDI, and uPDI	Using existing studies is efficient and is cost-effective by limiting need for new data	No need to complete new food surveys beyond those already included
Does not require *a priori* or self-reported dietary groupings (vegan, vegetarian, omnivore)	Reduces concerns over degree of adherence to a specific diet or dietary category	Obviates need to deal with vague and heterogeneous diet categories	Avoids categorizing diet patterns and related value judgments (e.g., ethical vegan)
Provides a continuous dietary index, not just a binary measure of adherence to a specific diet type	Allows comparisons by index extremes (quartiles, quintiles, deciles) and dose–response analysis	Guidance is facilitated by low versus high index level comparisons and by dose–response information	Comparisons of high versus low index outcomes are easy to grasp for healthy versus unhealthy plant-based foods
Shows benefits of healthy plant foods that might be missed by a PDI or diet category that does not consider plant-food quality	hPDI may better detect positive associations with outcomes than an overall PDI in a given sample (Tables 2–13)	Emphasizes healthy foods, not just foods in a specific diet or food group, allowing more nuanced dietary recommendations	Raises awareness about the benefits of eating healthy foods and why being “plant-based” does not ensure a high diet quality
Shows detriments of unhealthy plant foods that might be missed by a PDI or diet category that does not consider plant-food quality	uPDI may better detect negative associations with outcomes than an overall PDI in a given sample (Tables 2–13)	Highlights unhealthy refined and highly processed plant foods and beverages to avoid in nutrition guidelines	Raises awareness about the detriments of refined grains, fruit juices, sweets, sugar-sweetened beverages, and processed foods
Conceptualizes healthy eating as a continuum of food choices, not as strict adherence to a specific diet type, or as plant- versus animal-foods	Generalizability of findings is increased by seeing impact of quality changes and by aligning better with real-world diets	Guidance may promote better adherence if promoting healthy plant foods, rather than shunning animal or unhealthy foods	Empowers consumers to make incremental additions of healthy plant foods that may ultimately displace unhealthy foods

PDI, plant-based dietary index; hPDI, healthful PDI; uPDI, unhealthful PDI.

The inclusion of potatoes as a less healthy food in Table 1 is based on Satija and colleagues (4), who pioneered the concept of hPDI versus uPDI. We did not, however, assess the specific food components of the dietary indices in our included studies, so we do not know how specific investigators categorized potatoes. The association of potato consumption with health outcomes is controversial, with some pooled analyses of prospective studies finding a higher risk of hypertension or type 2 diabetes (112–114), but others showing no association with obesity, mortality, type 2 diabetes, or cardiovascular disease (115, 116). One study found a higher risk of type 2 diabetes with French fries, which was reduced by replacing potatoes with whole grains (112).

The animal foods in Table 1 do not distinguish by their potential health impact (healthy vs. less healthy) even though their inclusion in the diet adversely affects the plant-based dietary indices. Systematic reviews, however, have often shown adverse associations of omnivore diets with many of the health outcomes in our source articles, including obesity (117), type 2 diabetes (118), breast cancer (119), all-cause mortality (120), coronary artery disease (5, 121), inflammatory biomarkers (122), and cardiometabolic risk factors (123, 124). Similarly, the Global Burden of Disease Study found positive associations of high dietary trans fats, red meat, processed meat, and sugar-sweetened beverages with mortality from non-communicable diseases, but larger associations were found when the diet was low in healthy plant foods (whole grains, fruits, nuts, seeds, legumes, or vegetables) (109). Consuming fish and seafood have less consistent health associations compared with meat or plant foods (119, 120, 124), which is also the case for eggs and dairy products (125–127).

Strengths of our research include using *a priori* protocols for conducting and reporting the scoping review (13, 14), which is the first to systematically assess the contributions of hPDI and uPDI as correlates of health status. As recommended as a best practice when conducting a scoping review (14), we used dual, independent investigators to assess study eligibility and extract data, thereby reducing bias and improving accuracy. We contribute to understanding of how the quality of a plant-based diet can impact associations with health outcomes overall (Tables 2, 3), focusing on the novel concept of concordance versus discordance (Tables 3, 5–13), which has not been previously reported. We identified gaps in the existing knowledge base and provided perspective on the implications of our review findings for investigators, policy makers, and consumers (Table 14).

Limitations of our research, as for any systematic review, relate primarily to the breadth of available source articles. We used rigorous techniques, with dual investigators, to identify source articles in PubMed/MEDLINE, CINAHL, and EMBASE, but recognize that the subsequent Web of Science citation search was done post-hoc, which may have introduced bias, but is similar to checking source article bibliographies for additional relevant articles in a traditional systematic review. A scoping review does not include assessing study quality or pooling data with meta-analytic techniques (13), so we do not know the overall risk of bias or the level of heterogeneity in study protocols, outcome assessment, or results reporting. Our goal, however, was to help inform decisions and raise awareness about the importance of plant-based diet quality when interpreting evidence, in contrast to a systematic review for which risk of bias assessment is an inherent aspect of evidence synthesis.

Although all studies used the hPDI, uPDI, or both (or in a few cases the hPVD, uPVD, or both), there were some differences in how the indices were defined and calculated, even if based on the broad principles in Table 1. The general concept, however, of distinguishing healthy versus less healthy plant-based foods, is incorporated in most current scoring methods for assessing plant-based diet quality (128). Further, the extreme comparisons of index levels were based on varying thresholds, which included quartiles, quintiles, and deciles. We did not provide quantitative estimates of effect size (individual studies or pooled analyses), although this is more a limitation of scoping reviews, in general, than our specific research. Last, we do not know the generalizability of our findings to specific populations (e.g., pregnant or lactating women), but the included articles were often based on large population cohorts (see online Supplementary Appendix) that would support relevance to diverse subject groups.

Another limitation relates to assessing concordance (Table 3) using statistical significance as the primary determinant, and directionality of the association (positive vs. negative) as a secondary determinant for statistically significant associations. This could explain the relatively high levels of concordance between the hPDI and PDI (70.4%), and, to a lesser extent, between the hPDI and uPDI (47.7%), because the magnitude of effect size is not part of this determination. Although we purposefully did not report effect sizes, we did observe that they nearly always favored the hPDI in magnitude, even if not statistically significant (see individual study outcome data in the online Supplementary Appendix).

## Conclusion

Our findings, based on 95 included studies, demonstrate that distinguishing healthy versus less healthy plant foods in dietary indices can better detect significant associations with health outcomes than a single, overall plant-based dietary index. A high level of healthy plant food consumption was most often associated with favorable outcomes for obesity, mortality, diabetes, cardiovascular disease, and psychiatric disorders, whereas a high level of less healthy plant food consumption was most often associated with unfavorable outcomes for obesity, mortality, psychiatric disorders, and cardiometabolic risk factors. When there were discordant associations for the hPDI compared to the uPDI or PDI, the findings always favored the hPDI over the uPDI, and nearly always favored the hPDI over the PDI.

These results, combined with the implications of healthy plant food consumption for researchers, policy makers, and consumers (Table 14), suggest that the current global trend of rapid growth in related research and publications is likely to continue. Future research should incorporate measures of diet quality when assessing the association of plant-based diets with health outcomes. With increasing reporting and standardization of plant-based indices that adjust for diet quality, we anticipate a blossoming number of systematic reviews and meta-analyses that will assist guideline developers and policy makers in making informed, evidence-based recommendations.

## Author contributions

RR conception and design of the work, data analysis and interpretation, drafting of the work, approval for publication of the content, accountability for all aspects of the work. HJ and MW design of the work, data acquisition, critical revision of content, approval for publication of the content, accountability for all aspects of the work. All authors contributed to the article and approved the submitted version.

## Conflict of interest

The authors declare that the research was conducted in the absence of any commercial or financial relationships that could be construed as a potential conflict of interest.

The handling editor AB declared a shared committee American College of Lifestyle Medicine with the authors RR at the time of review.

## Publisher’s note

All claims expressed in this article are solely those of the authors and do not necessarily represent those of their affiliated organizations, or those of the publisher, the editors and the reviewers. Any product that may be evaluated in this article, or claim that may be made by its manufacturer, is not guaranteed or endorsed by the publisher.
